# Node Deployment with *k*-Connectivity in Sensor Networks for Crop Information Full Coverage Monitoring

**DOI:** 10.3390/s16122096

**Published:** 2016-12-09

**Authors:** Naisen Liu, Weixing Cao, Yan Zhu, Jingchao Zhang, Fangrong Pang, Jun Ni

**Affiliations:** 1 National Engineering and Technology Center for Agriculture, Jiangsu Key Laboratory for Information Agriculture, Collaborative Innovation Center for Modern Crop Production, Jiangsu Collaborative Innovation Center for the Technology and Application of Internet of Things, Nanjing Agriculture University, Nanjing 210095, China; boomzip@163.com (N.L.); caow@njau.edu.cn (W.C.); yanzhu@njau.edu.cn (Y.Z.); pangfangrong@njau.edu.cn (F.P.); 2 Jiangsu Key Laboratory for Eco-Agricultural Biotechnology around Hongze Lake, Jiangsu Collaborative Innovation Center of Regional Modern Agriculture & Environmental Protection, Huaiyin Normal University, Huai’an 223300, China; 3 Nanjing Institute of Agricultural Mechanization of National Ministry of Agriculture, Nanjing 210014, China; zhangjc9@163.com

**Keywords:** node deployment, *k*-connectivity, full coverage, communications silos, farmland scale, crop information, genetic algorithm

## Abstract

Wireless sensor networks (WSNs) are suitable for the continuous monitoring of crop information in large-scale farmland. The information obtained is great for regulation of crop growth and achieving high yields in precision agriculture (PA). In order to realize full coverage and *k*-connectivity WSN deployment for monitoring crop growth information of farmland on a large scale and to ensure the accuracy of the monitored data, a new WSN deployment method using a genetic algorithm (GA) is here proposed. The fitness function of GA was constructed based on the following WSN deployment criteria: (1) nodes must be located in the corresponding plots; (2) WSN must have *k*-connectivity; (3) WSN must have no communication silos; (4) the minimum distance between node and plot boundary must be greater than a specific value to prevent each node from being affected by the farmland edge effect. The deployment experiments were performed on natural farmland and on irregular farmland divided based on spatial differences of soil nutrients. Results showed that both WSNs gave full coverage, there were no communication silos, and the minimum connectivity of nodes was equal to *k*. The deployment was tested for different values of *k* and transmission distance (d) to the node. The results showed that, when *d* was set to 200 m, as *k* increased from 2 to 4 the minimum connectivity of nodes increases and is equal to *k*. When *k* was set to 2, the average connectivity of all nodes increased in a linear manner with the increase of *d* from 140 m to 250 m, and the minimum connectivity does not change.

## 1. Introduction

Real-time, large-scale assessment of crop conditions is conducive to precise control of crop growth and development [1,2,3,4]. It has important guiding significance in increasing production and improving crop quality. Precision agriculture uses information technology to realize the efficient management of agriculture according to the temporal and spatial differences in farmland and crop growth [5,6]. These differences include soil nutrients, soil water, crop biomass, and chlorophyll a content. This method can be used to estimate, assess, and understand these differences in order to determine irrigation and fertilizer requirements, production and ripening phases, and optimum sowing and harvesting times [7]. Accurate spatial and temporal variance of farmland is of great significance to the implementation of precision agriculture. The traditional field crop sampling and laboratory analysis method involves considerable time lag and so cannot meet the needs of modern crop production and management. With the development of quantitative spectral analysis techniques, the relationships between crop canopy reflectance spectra and crop growth information have been assessed [8,9,10], and the monitoring models of different crop information such as yield, biomass, nitrogen content, and chlorophyll a content were constructed based on crop spectra [11,12,13,14,15,16]. The development of spectrum monitoring technology for crop growth information promotes the development of crop sensors and portable crop growth monitors [17,18,19,20,21], thus providing an effective means of obtaining crop growth information in real time, using sensors installed in wireless sensor networks (WSNs), intelligent agricultural machinery, and unmanned aerial vehicles (UAVs) [2,22,23,24,25,26]. Agricultural production covers broad areas, highly variegated field information spaces, and long crop cycles, which increases the difficulty of obtaining crop information in real time. WSNs offer low cost, low power consumption, and the information can be transmitted wirelessly [27]. They can achieve long-term monitoring of the target area by deploying a large number of nodes in the area [28,29].

WSN has been widely used in many fields, such as military reconnaissance [30], monitoring of biological habitats [31], environmental monitoring [32,33], intelligent transportation [34], and health care [35]. In agriculture, WSNs were used to monitor environmental parameters such as temperature, humidity, and illumination [36]. They are also used to monitor crop growth information. Bauer et al. integrated photosynthetically active radiation (PAR) sensors into WSNs to achieve in-situ measurement of crop leaf area index, and the measurement accuracy was closely correlated with LAI-2200 measurements [22]. Liu et al. used WSN equipped with spectral sensors to monitor the growth information of rice and wheat [2].

WSN monitoring capabilities and data acquisition capabilities depend on the node deployment status within the network. Node deployment is mainly related to features such as coverage, connectivity, cost, and network lifetime [37,38,39,40,41,42], wherein coverage and connectivity are the most important indicators in the assessment of the performance of WSN information collection systems [43]. The goal of full coverage node deployment is to access network target area information without missing any relevant data. Due to temporal and spatial differences in crop growth information, therefore, the primary issue in crop WSN deployment should be to fully access crop information. According to the correlation between crop growth and farmland soil, Liu et al. used differences in the spatial distribution of soil nutrients to divide large-scale farmland into subsections [2]. After division, soil nutrients within each divided subsection were similar, so deploying one crop sensor per field allowed full coverage monitoring of crop information, which breaks the blindness of farmland sensor node deployment and greatly reduces its cost. Large farms are composed of many plots. If the spatial variability of plot is great, then the farmland could be divided using method proposed by Liu et al. [2]. However, if the spatial variability of plot is small, then the crop information would be almost the same. The goal of node connectivity deployment is that the network must reliably transmit information from the target region to the sink node. Because the arable land area is very wide, the key issue of crop WSN deployment is reliable transmission of information. According to the connectivity of sensor nodes, WSN can be divided into star, mesh, and hierarchical networks [44,45,46,47]. Due to the size and power limitations of hardware devices, WSN nodes can only transmit information over short distances. Star networks are not suitable for large-scale deployment across wide areas of farmland. Meanwhile in order to achieve full network connectivity, gateway devices are generally placed in the spatial center of all node locations, which limits the applicability of this kind of deployment in applications. The nodes in the mesh network transmit information to the sink node in a multi-hop manner, so this networking mode is more suitable for large-scale WSNs [48]. Node information transmission in the form of multi-hop addresses the restriction of node transmission distance relatively well and is somewhat compatible with large-scale deployment on large parcels of farmland. However, there are large numbers of relay nodes in the network, which increases the redundancy of monitoring information and increases deployment costs. Optimizing WSN coverage and connectivity are both restrictive objectives, and they ensure reliable transmission of information under the premise of full coverage monitoring by the WSN. This forms a bottleneck for sensor nodes regarding large-scale deployment across large areas of farmland, an issue that urgently needs to be resolved.

In this paper, two kinds of monitoring specific to the needs of large-scale agricultural and natural plots, full-coverage, and *k*-connectivity WSN deployment method were studied on farmland. Large parcels of farmland were subdivided into many plots based on the spatial distribution of soil nutrients [2]. After division, soil nutrients were confirmed to be similar across each plot. The crop information can be fully monitored by deploying one node in each plot. A genetic algorithm (GA) was used to optimize the positions of the nodes to achieve robust crop WSN connectivity deployment. This deployment method ensured that the farmland WSN would provide full coverage and *k*-connectivity, have no communication silos, and that the nodes deployed would not be impacted by the farmland edge effect, improving the accuracy of WSN monitoring crop growth information.

## 2. Farmland Full Coverage Monitoring and WSN *k*-Connectivity Deployment Method

Large farmland areas by definition cover a vast territory and they are subject to considerable variation in soil nutrient levels and may have uneven crop growth. In order to collect comprehensive crop growth information in the field, the farmland must be divided into subsections within which soil nutrient levels remain similar. Then one node is deployed for each section of the divided field to facilitate the collection of comprehensive crop growth information in the field. In natural fields, since there is correlation between spatial distribution of soil nutrients, and consistent agricultural measures, crop growth tended to be the same within each field, so sensor node deployment in natural fields can also realize full-coverage deployment of the crop information monitoring network. Full coverage requires that all WSN nodes should be deployed within the corresponding field. For example, node 1 should be deployed in field 1, node 2 should be deployed in field 2, and so on. Plant growth requires solar radiation, carbon dioxide, soil moisture, nutrients, and other resources, when the resources in different fields are basically the same, the differences in crop growth will be small; if not, the differences may be great. However, plants that grow close to the edges of a given plot may have access to difference resources, such as extra solar radiation or soil moisture, so their growth state may be different from that of plants in the middle of the field [49]. To ensure the accuracy of measurement, the sampling points should be positioned in areas that appear representative of the majority of the crop growth in that designated area, so the sensor nodes should be deployed far from field boundaries. Previous deployments of farmland WSN did take this issue into account. Robust network connectivity requires that each node have at least *k* transmission paths, that is, the network is *k*-connected [50,51], *k*-connection can effectively improve the robustness of network connectivity (*k* ≥ 2) [52,53,54], when energy is depleted or part node fails, other nodes can ensure normal WSN communication. In the case of *k*-connectivity, WSN should not have internal communications silos, to ensure that information from any node can be transferred to gateway devices without failure. In this way, full-coverage WSN monitoring of farmland connectivity deployment is a multi-objective optimization problem. Any such system must meet the following criteria: (1) WSN nodes must be located in the corresponding field; (2) The WSN must be *k*-connected; (3) The WSN should not have any internal communication silos; (4) The distance of sensor nodes to field boundaries should be greater than a certain value. WSN network deployment with multiple optimized objectives has proven to be an NP-complete problem (non-deterministic polynomial complete problem) [55,56,57]. The GA was here used to convert farmland full coverage monitoring WSN connectivity deployment rules into a single objective, simplify constraint relationships between sensor nodes, and optimize node position to determine wide-area farmland full-coverage monitoring WSN network topology.

## 3. Avoiding the Farmland Edge Effect on the Nodes Deployed

**Figure 1:**
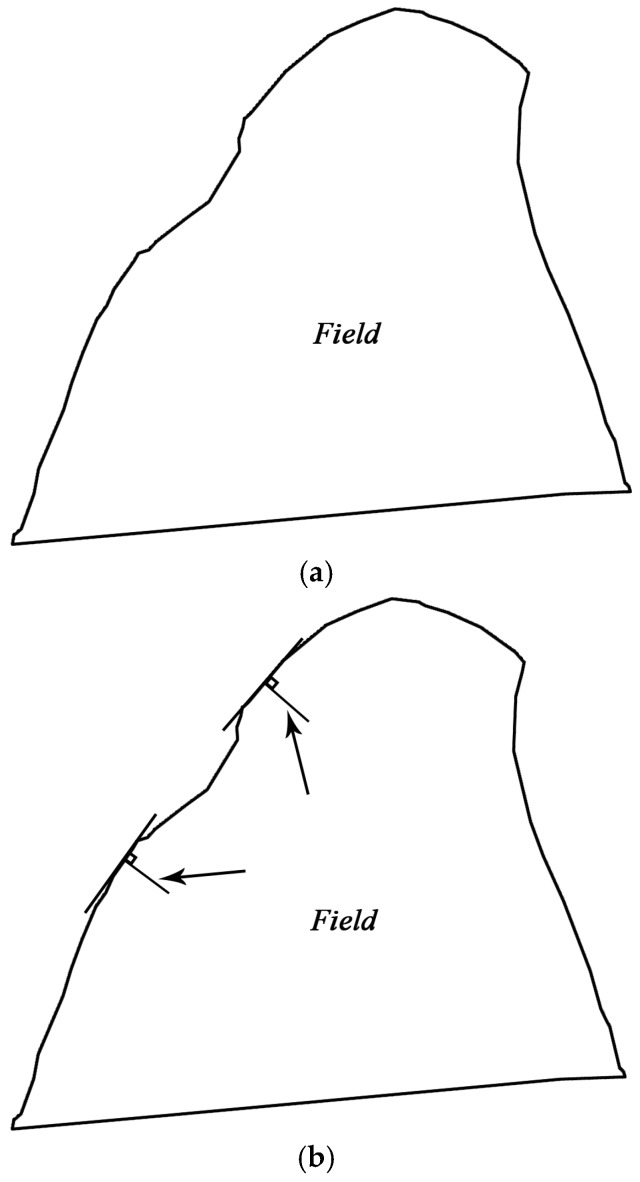

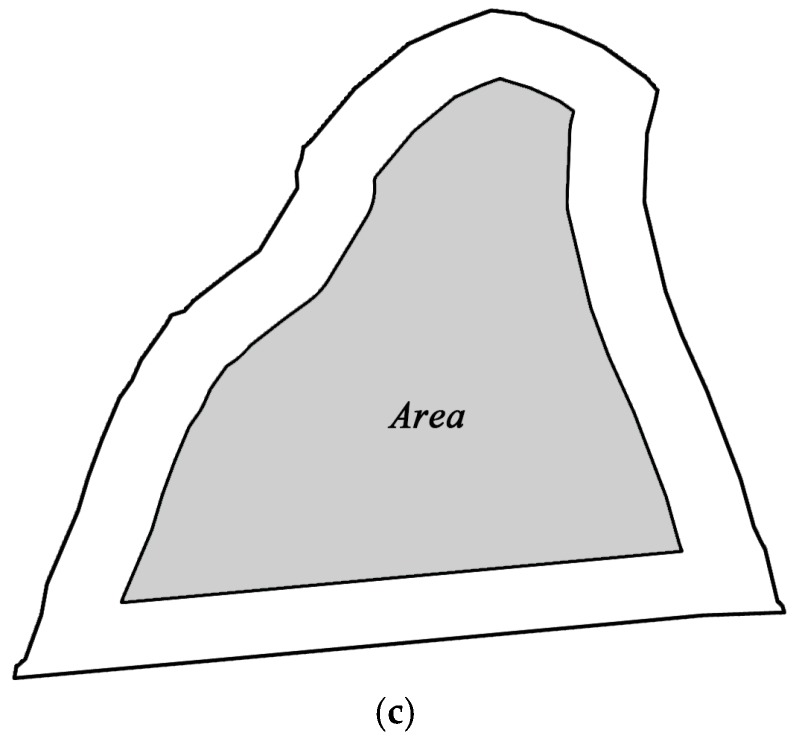
Schematic diagram showing the establishment of the area in which nodes must be deployed from farmland. (**a**) Field; (**b**) Vertical segment drawn from the tangent of the border of the field; (**c**) Node deployment area.

Due to the edge effect of farmland, the growth state of crops close to the boundaries of the plots examined is different from that of plants grown near the middle of each plot [49]. It is very important that crop sensor nodes are deployed at a distance from field boundaries to ensure the accuracy of network monitoring crop growth information. Bell et al. determined that, to avoid the edge effect, sampling points should be at least 50 cm away from the boundary of the farm plot, and the distance should be increased in cases of soil stress [58]. Fischer suggested the distance should be between 25 cm and 100 cm, depending on crop height [49]. In this way, the distance between nodes and field boundary should be at least 1 m to ensure the accuracy of the monitoring of crop growth information. When nodes are deployed using GA, the fitness function is used to determine whether the minimum distance between the node and the plot boundary is too small. However, the calculation of the fitness of GA needs a lot of time. For every iteration of GA, each fitness value of each individual in the population must be calculated, and each individual has many nodes, so a very large number of nodes is needed to calculate the distance, and this requires a lot of time, thus affecting the time-consuming performance of WSN deployment method. In order to improve the operating efficiency of GA, the 4th deployment principle was specially handled. The method used is as follows:

When a sensor node is deployed as in the field shown in Figure 1a, let the distance between node and boundary be greater than *x*, draw a vertical segment with length *x* from the tangent of the field boundary, i.e., draw normal boundary with length *x* inside the field (Figure 1b), connect the vertices of line segments, and remove the cross-section to designate the enclosed area (Figure 1c). The distance from any point within the area to the boundary is greater than *x*. The deployment of sensor nodes within the area will meet principle (4): the distance between node and field boundary should be greater than a certain value. It also meet the deployment principles (1): all WSN nodes must be located within the corresponding fields.

## 4. Genetic Algorithm for Farmland Full-Coverage Monitoring WSN Connectivity Deployment

Farmland full coverage monitoring WSN connectivity deployment is a NP-complete problem [59,60,61]. In computational complexity theory, if problem can be solved in polynomial time, then this is called a P problem. NP denotes the set of all problems solvable by a non-deterministic polynomial time algorithm. NP problems are considered “hard” in the sense that they are not currently solvable in deterministic polynomial time. A decision problem is NP-complete when it is both in NP and NP-hard [62]. Evolutionary algorithms are effective means of solving NP problems [63,64]. These algorithms simulate the mechanism of biological evolution. They are mature global optimization methods, highly robust and broadly applicable. They are self-organizing, adaptive, and self-learning, not restricted by the nature of the problem. They can deal effectively with complex problems that are difficult to solve using traditional optimization algorithms [65]. Evolutionary algorithms have been widely used in WSN deployment projects, such as simultaneous optimization of network coverage and energy consumption, connectivity and energy consumption [52], and coverage, connectivity, and energy consumption [66]. GA one of evolutionary algorithms was used to optimize node positions in order to meet the principles of network deployment in this paper.

**Figure 2:**
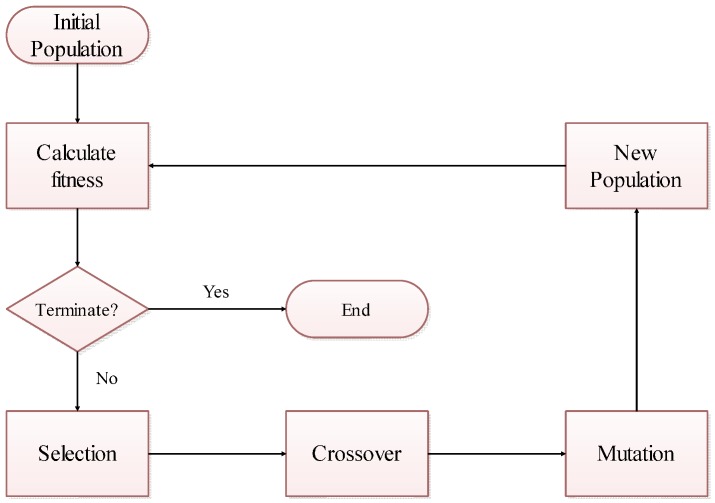
Flowchart of the genetic algorithm.

GA is a random global search and optimization method developed from simulation of natural biological evolution mechanism; it does not require derivation or continuity functions for the problem to be optimized. It automatically acquires and accumulates knowledge regarding searching space in the search process and self-adapt and control search processes in order to find the optimal solution. Each possible optimization solution is represented by a simple string called chromosome or individual. A population of chromosomes is generated when GA is initialized. The quality of chromosome is evaluated using a fitness function. After initializing the population, GA performs iterative operations and exits when the iteration termination conditions is reached, such as reaching the maximum number of iterations or finding an excellent solution. In each iteration, GA performs four operations in sequence: fitness calculation, selection, crossover, and mutation. The GA operation flowchart is shown in Figure 2.

### 4.1. Chromosome Encoding Representing Network Node Locations

**Figure 3:**
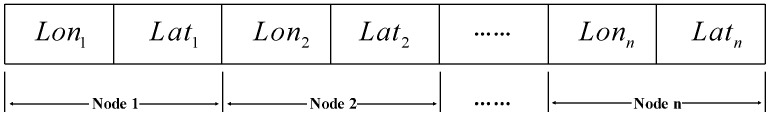
Schematic diagram of real-number chromosome encoding in WSN deployment. Note: *Lon_i_* and *Lat_i_* represent the longitude and latitude of node *i*, respectively.

Chromosome encoding involves converting feasible solutions of a specific problem from their solution space to the space that the GA can handle. Chromosome can be encoded in both binary and real numbers. Binary is discrete data, so its encoding will lose accuracy, while the real number encoding does not have this problem [67], so it was selected to encode the chromosome. The longitude and latitude of all nodes were sequentially connected to generate chromosomes, as shown in Figure 3.

**Figure 4:**
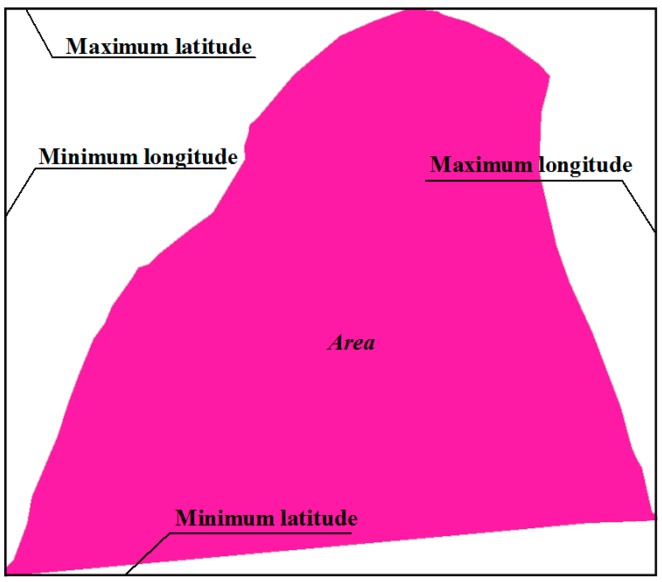
Schematic of the value range in chromosome.

At initialization, chromosome value is randomly generated. If the ranges of *Lon_i_* and *Lat_i_* are defined appropriately, the GA searching space can be reduced in size and the algorithm’s optimization efficiency can be improved. The node deployment region is here called *Area* (Figure 4). The minimum rectangle was used to frame the *Area*, the minimum and maximum longitude of the rectangle were the range of *Lon_i_*, and the minimum and maximum latitudes of the rectangle were the range of *Lat_i_*, as shown in Figure 4.

### 4.2. Selection, Crossover and Mutation Operations

The selection operation involves simulating a survival of the fittest mechanism in biological evolution to select the best individuals from the population, based on individual fitness. Commonly used algorithms in the selection operation include roulette wheel selection, tournament selection, stochastic universal selection, and similar processes. The roulette wheel selection, known as the selective Monte Carlo method, was selected as selection operation of this paper. It involves randomly selecting the best individuals according to their fitness degree.

In nature, creatures produce new individuals through recombination of homologous chromosomes, and recombination is an important part of genetics and evolution. Crossover is a simulation of biological recombination. It involves randomly selecting two individuals with a certain probability from the individuals selected by selection operation, and exchanging information to produce offspring. The crossover probability generally ranges from 0.40 to 0.99. There are several methods of crossover operation, and the linear recombination algorithm was selected since the chromosome is real number encoded.

Mutation operations change the information of the offspring with small probability. They play a specific role in maintaining genetic diversity and prevent the genetic algorithm from converging prematurely [68,69,70]. Mutation probability generally ranges from 0.0001 to 0.1. In this paper, the real value of variation was used for mutation operations, and its calculation method is shown in Equation (1):

(1) X'=X±0.5RΔ

Here, *X*’ is the value after mutation, *X* is the value before mutation, $\Delta =\sum_{i=0}^{m}\frac{a(i)}{2^{i}}$, a(i) is taken as 1 with probability of $\frac{1}{m}$, taken as 0 with probability of $1-\frac{1}{m}$, usually taken as m=20, *R* is the value range of variables.

### 4.3. Construction of the Fitness Function for WSN Deployment

Fitness was used to characterize individual quality in order to measure the outstanding level at which the individual would reach or approach the optimal solution or help to find the optimal solution. Individuals with high fitness will have more opportunities to produce offspring. The fitness function should be associated with the problem to be optimized. It plays a vital role in the ability of GA to solve practical problems. For multi-objective optimization problems, there are several ways of establishing fitness functions [71,72]. In these methods, converting multi-objective optimization problems into single objective optimization is a common and effective method. For a multi-objective optimization problem, if empowering weight $w_{i}(i=1,2,\ldots ,n)$ to each sub-objective function $f_{i}(x)(i=1,2,\ldots ,n)$, linear weight and *u* of each sub-objective function can be found. As shown in Equation (2), *u* can be used as the fitness value of multi-objective optimization problems:

(2) $$u=\sum_{i=1}^{n}w_{i}\cdot f_{i}(x)$$

In this paper, there are a total of four WSN deployment principles, and principle (4) can be converted to principle (1) by processing (Section 3).

**Figure 5:**
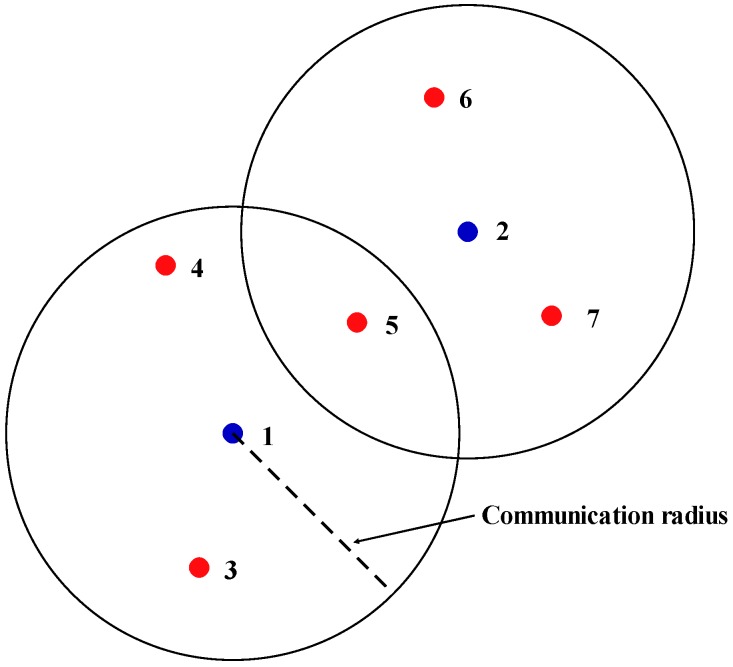
Schematic of neighbor nodes. The neighbor nodes of node 1 were node 3, node 4, and node 5. The neighbor nodes of node 2 were node 5, node 6, and node 7.

The term “*k*-connected network” refers to networks in which any node has at least *k* neighbor nodes. Let the distance between two nodes be *d*. The node communication distance is *d_com_*, and nodes that meet *d* < *d_com_* are considered neighbor nodes. That is, neighbor nodes are the other nodes within the communication radius, as shown in Figure 5. In this way, the number of neighboring nodes was used to calculate the connectivity of a single node. Distance between nodes can be calculated according to Equation (3):

(3) $$d=2R\arcsin(\sqrt{h})$$

Here, *R* is the radius of the Earth, which averages 6371 km. *h* can be obtained by calculation through Equation (4):

(4) $$h=\mathrm{haversin}(\phi_{2}-\phi_{1})+\cos(\phi_{1})\cos(\phi_{2})\mathrm{haversin}(lon_{2}-lon_{1})$$

Here, $\phi_{1}$ and $\phi_{2}$ are the latitude and longitude of two points, respectively, $lon_{1}$ and $lon_{2}$ are the longitudes of two points. See Equation (5) for function haversin(θ):

(5) $$\mathrm{harversin}(\theta )=\sin^{2}(\theta /2)=(1-\cos(\theta ))/2$$

**Figure 6:**
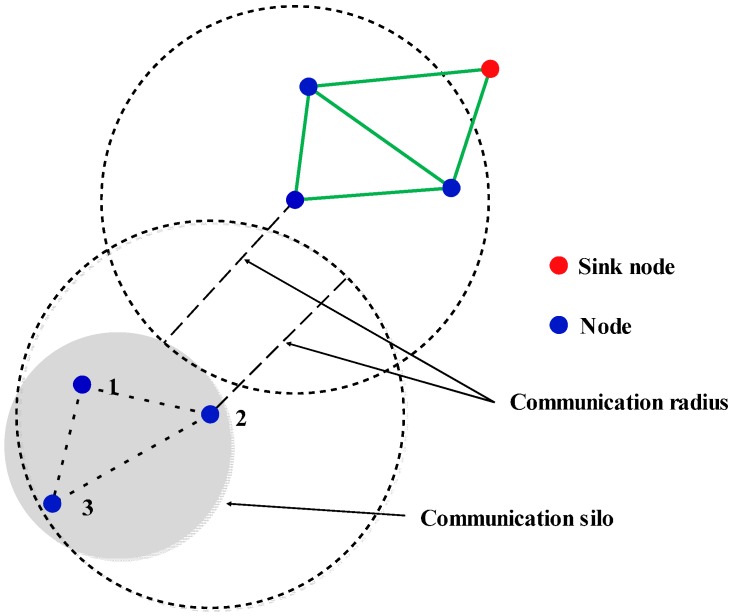
There was a communication silo in this *k*-connected network.

In the case the network is *k*-connected (e.g., *k* = 2), some nodes may still be off-network, their information cannot be transmitted to sink node, as shown in Figure 6. As shown in Figure 6, node 1, node 2, and node 3 are neighboring nodes. That is, each of them have two neighboring nodes, but their information cannot be transferred to the sink node and become information silos.

**Figure 7:**
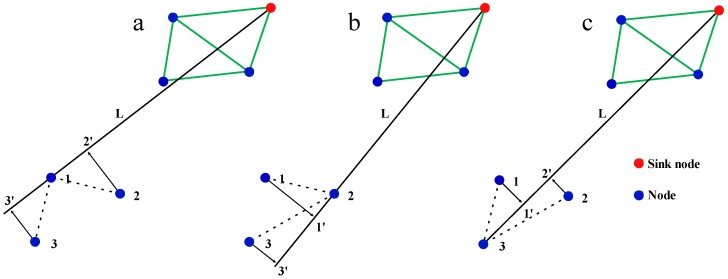
The direction of transmission of node information. (**a**) Nodes 2 and 3 neighbored node 1. The projection position of node 2 is 2’, and 2’ is between sink node and node 1. The projection position of node 3 is 3’ and it is on the extension line L; (**b**) Neighbor nodes of node 2 are node 1 and node 3. The projection positions of node 1 and node 3 are 1’ and 3’, the 1’ and 3’ are both on the extension line L; (**c**) Neighbor nodes of node 3 are node 1 and node 2. The projection positions of neighbor nodes were 1’ and 2’, the 1’ and 2’ are both between sink node and node 3.

The straight lines L are drawn from the sink node to node 1, node 2, and node 3, and projections of neighbor nodes are drawn on L. As shown in Figure 7a, the projection positions of neighbor node 1 on L were 2′ and 3′, respectively. Here, 2′ is between sink node and node 1, 3′ is on the extension line, which indicates that if node 1 transmits information to node 2, the distance (*D*) between information position and the sink node will be shorter. However, if information is transmitted from node 1 to node 3, *D* will be longer. As shown, that path between node 1 and node 2 is more conducive to information transmission from node 1. The projection situation of neighbor nodes of node 2 is shown in Figure 7b. The projection positions of neighbor nodes 1′ and 3′ are both on an extension of the connection line between sink node and node 2, indicating that regardless of communication with any neighbor nodes, information from node 2 is transmitted away from the sink node. As shown in Figure 7c, the projection positions of neighbor nodes are both between the sink node and node 3, which indicates that all the information transmitted from node 3 will be closer to the sink node. Based on the above analysis, the connectivity of node 3 is most conducive to information transmission, that of node 1 is second, and the communication situation of node 2 is the worst, which is not conducive to the transmission of information. The reason of node 1, node 2, and node 3 becoming information silo is that the connectivity of node 2 is not conducive to network information transmission (Figure 7).

**Figure 8:**
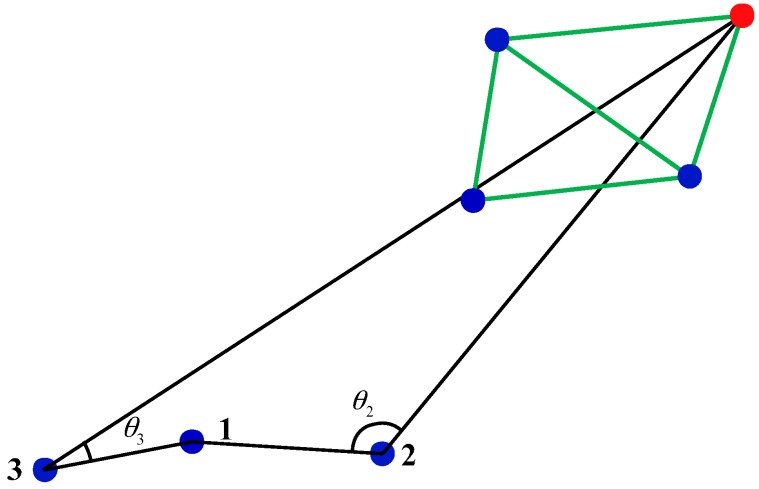
Schematic diagram of the node connectivity angle.

**Figure 9:**
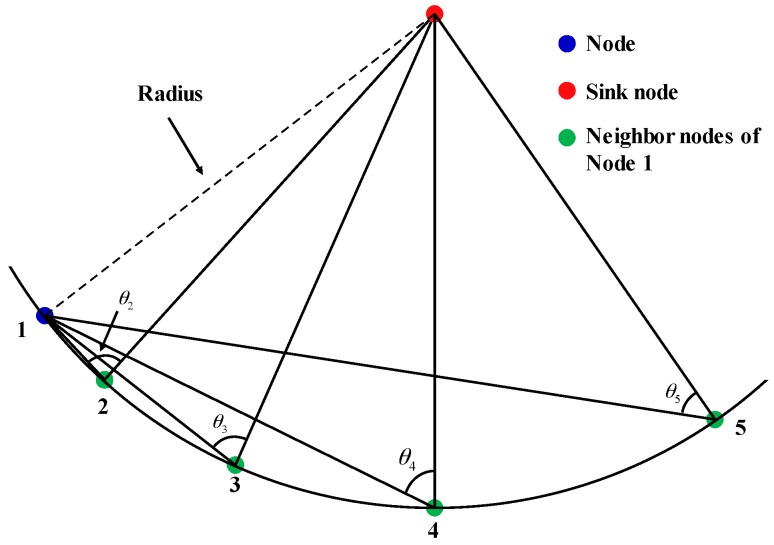
Node and neighbor nodes are on circumference of a circle, and the sink node is in the center of the circle.

It is advantageous for network information transmission if the projection positions of neighbor nodes are on a line connected a node to the sink node. It is unfavorable for transmission if the projection positions are on the extension of the connection line. The impact of neighbor node location on information transmission can be measured using the connectivity angle. Here the connectivity angle is defined as the angle between two lines, which are the connection between the node and the neighbor and the connection between the neighbor node and the sink node, as shown in Figure 8. When in communication with node 2, the connectivity angle of node 1 is *θ*_2_, and when in communication with node 3, the connectivity angle is *θ*_3_. The maximum value of the connectivity angle is 180°, and the minimum value is 0°. When the connectivity angle is 180°, the neighbor node is on the connection line of sink node and the node. When connectivity angle is 0°, neighbor node is on the extension line of connection line between the sink node and the node. When the connectivity angle is large, the distance to sink node becomes small after the information is transmitted, preventing the emergence of information silos. However, there are also exceptional circumstances. In this work, when the node and its neighbor node/nodes are on the circumference of a circle centered by a sink node, although connectivity angle changes, the distance between the neighbor node and sink node did not change (Figure 9), but the probability of this happening is extremely low, and this change has almost no effect on the operation of GA and could therefore be disregarded.

Based on the discussion given above, the fitness function of WSN deployment is defined as follows:

(6) $$Fitness=a\times Fitness_{\mathrm{in}-\mathrm{area}}+b\times Fitness_{k-\mathrm{conn}}+c\times Fitness_{\theta }$$

Here, $Fitness_{in-area}$, $Fitness_{k-conn}$, and $Fitness_{\theta }$ represent the score of nodes located within corresponding fields, score of node connectivity number, and score of node connectivity angle. $Fitness_{\mathrm{in}-\mathrm{area}}\in [0,1]$, $Fitness_{k-\mathrm{conn}}\in [0,1]$, $Fitness_{k-\mathrm{conn}}\in [0,1]$, 0<a<1, 0<b<1, 0<c<1, a+b+c=1:

(7) $$Fitness_{\mathrm{in}-\mathrm{area}}=\frac{1}{n}\sum_{i=1}^{n}F_{i_{\mathrm{in}-\mathrm{area}}}$$

Here, *n* is the number of fields, and also the number of nodes. $F_{i_{in-\mathrm{area}}}=\left\{ \begin{array}{l} 1,\mathrm{node}\;i\;\mathrm{is}\;\mathrm{located}\;\mathrm{inside}\;\mathrm{field}\;i\\ 0,\mathrm{node}\;i\;\mathrm{is}\;\mathrm{located}\;\mathrm{outside}\mathrm{field}\;i \end{array}\right.$.

(8) $$Fitness_{k-\mathrm{conn}}=\frac{1}{n+1}\left(\sum_{i=1}^{n}F_{i_{k-\mathrm{conn}}}+F_{sn_{k-\mathrm{conn}}}\right)$$

wherein *n* is the number of nodes, $F_{i_{k-\mathrm{conn}}}=\{\begin{array}{ll} 1, & N_{neighbor}\geq k\\ 0, & N_{neighbor}<k \end{array}$, $N_{neighbor}$ is the number of neighbor nodes of node *i*, *k* is the connectivity number to be met by the network deployment. $F_{sn_{k-\mathrm{conn}}}=\left\{ \begin{array}{l} 1,\mathrm{number}\;\mathrm{of}\;\mathrm{neighbor}\;\mathrm{nodes}\;\mathrm{of}\;\mathrm{sink}\;\mathrm{node}\geq k\\ 0,\mathrm{number}\;\mathrm{of}\;\mathrm{neighbor}\;\mathrm{nodes}\;\mathrm{of}\;\mathrm{sink}\;\mathrm{node}<k \end{array}\right.$.

(9) $$Fitness_{\theta }=\frac{1}{N}\sum_{i=1}^{N}{\left(\frac{\theta_{i_{\max}}}{180}\right)}^{2}$$

Here, $N=n-n_{sn}$, *n* was the number of nodes, $n_{sn}$ was the number of neighbor nodes of sink node. $\theta_{i_{\max}}$ was the maximum angle among *k* connectivity angles of node *i*.

The values of *a*, *b*, and *c* are determined by using the analytic hierarchy process (AHP) [73]. This method involves breaking down the factors associated with decision-making. Quantitative analysis of decision-making is conducted on this basis. During the implementation of AHP, a judgment matrix based on the actual situation must be constructed using to the results of pairwise comparison (standards of pairwise comparisons are shown in Table 1). In this paper, $Fitness_{k-\mathrm{conn}}$ and $Fitness_{\theta }$ are considered equally important. $Fitness_{\mathrm{in}-\mathrm{area}}$ is more important than $Fitness_{k-\mathrm{conn}}$ and $Fitness_{\theta }$. These statuses were used to construct the judgment matrix after the AHP operation was performed. This produced a=0.7778, b=0.1111, and c=0.1111.

### 4.4. Multiple Population Evolution

**Figure 10:**
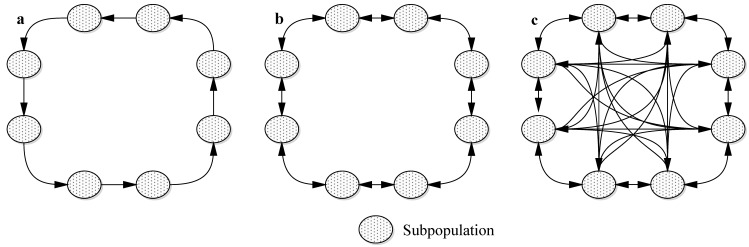
Migration topology of multiple population evolution. (**a**) Ring migration topology; (**b**) Neighborhood migration topology; (**c**) Unrestricted migration topology.

During the operation of classical GA, premature convergence can take place, and GA stops at a local optimum. To solve the premature convergence problem of GA, researchers have performed many studies [74,75,76], wherein multiple populations evolved independently, and appropriate exchanges of chromosomes (individuals) are made among populations after the evolution of certain numbers of generations, which can greatly improve the global optimization capabilities of GA [77,78]. Common ways of exchanging individuals among populations include ring migration, neighborhood migration, and unrestricted migration [79,80] (Figure 10). Ring migration occurs only between individuals in directly adjacent populations, and the migration is unidirectional. Neighborhood migration also takes place between directly adjacent populations, but the migration is bidirectional. Unrestricted migration can occur among any group of populations.

## 5. Development of Wireless Sensor Network Deployment Software

**Figure 11:**
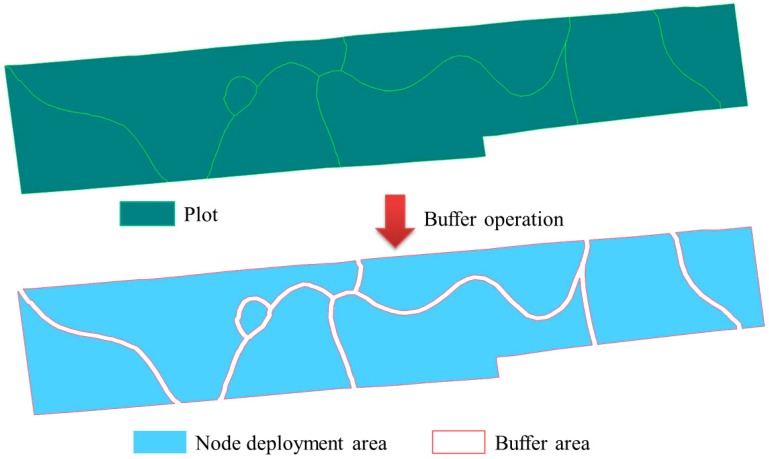
The diagram of generating node deployment area from farmland by GIS buffer operation.

The fourth WSN deployment principle in this paper is the distance between sensor nodes and field boundaries should be greater than a certain value. The principle could be fully meted by buffer operation in geo-information system (GIS). Here, an open-source software of OGR was used to implement the buffer operation. A node deployment area generated from field by this method is shown in Figure 11. The farmland is located in Rugao, Jiangsu Province, China, covering about 5 hectares, which was divided into eight fields by differences in the spatial distribution of soil nutrients [2]. The minimum distance between nodes and boundaries was set at 2 m.

**Figure 12:**
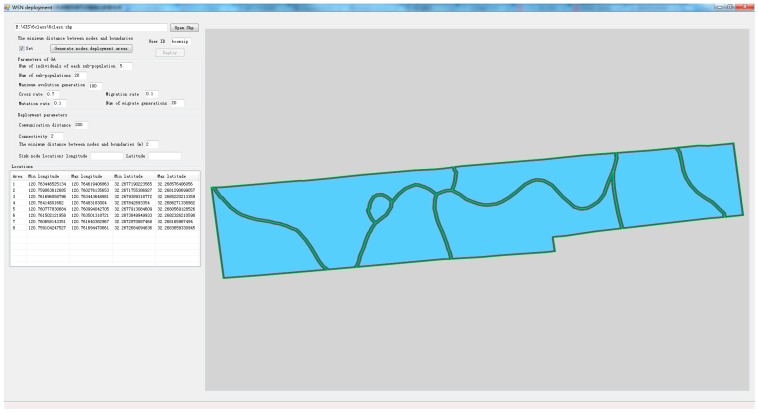
The interface of WSN deployment software.

WSN deployment software was compiled in the .NET framework using C# language based on the network deployment method in this paper. The genetic algorithm toolbox developed at Sheffield University was used. Whether a node is within the field or not, open-source software OGR was used to judge and SharpMap control was used to display network deployment results. The interface of WSN deployment software is shown in Figure 12.

## 6. Deployment of WSN for Monitoring Crop Growth Information

### 6.1. The CGMD302 Crop Growth Information Sensor

**Figure 13:**
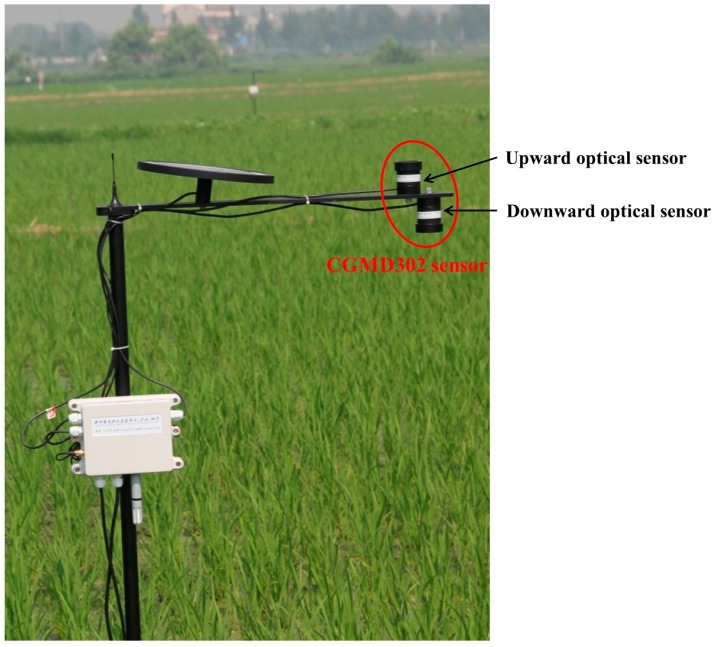
Field application of CGMD302 crop growth information sensor.

The WSN deployment method in this paper has no specific requirements regarding crop growth information sensors. To test the performance of the deployment method, a crop growth information sensor named CGMD302 was selected. The sensor was developed by the National Engineering and Technology Center for Information Agriculture at China’s Nanjing Agricultural University. It can obtain a variety of crop growth information, such as leaf area index (LAI), nitrogen content, biomass, and other factors. The sensor is made up of two kind of detection lenses (720 nm and 810 nm), which are used to detect canopy reflectance spectrum of a given crop. The sensor uses filters to split light using sunlight as its light source, and it is composed of upward and downward optical sensors. The upward optical sensor receives solar radiation at 720 and 810 nm, while the downward optical sensor receives reflected radiation of the crop canopy at the corresponding bands. The CGMD302 sensor is small and light, packaged with a cylindrical aluminum shell, with an aperture of 38 mm and a height of 50 mm. therefore, it is suitable for field application (Figure 13). On the WSN applications of monitoring crop growth information, each node can be connected to several different types of sensors, if a large amount of crop growth information needs to be monitored, and a single sensor cannot obtain all the information.

### 6.2. WSN Deployment on Irregular Farmland Divided by Differences of Spatial

**Figure 14:**
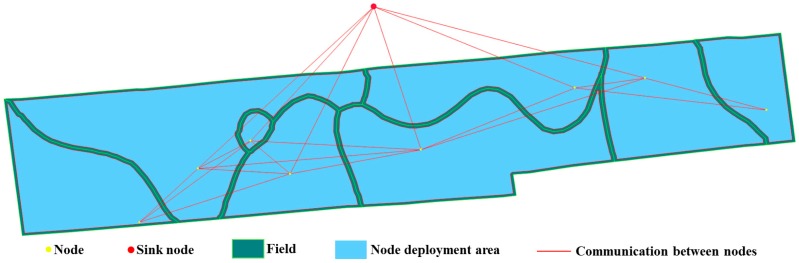
The WSN deployment on irregular farmland located in Rugao, Jiangsu Province, China. The communication distance is 200 m, and the connectivity is 2.

The WSN deployment software developed of this paper was used to deploy nodes on the plot of farmland shown in Figure 11, the farmland was divided into eight irregular plots according to the soil nutrient attributes by Liu et al. [2]. The minimum distance between nodes and field boundaries was at least 2 m, and the network connectivity was set to 2. There were 10 sub-populations of GA, and 100 individuals in each sub-population. The maximum evolution generation was 100 (iteration stop condition), the cross rate was 0.7, and the mutation rate was 0.1. Individuals were allowed to migrate without restriction once among sub-populations every 10 generations, and the migration rate was 0.1. Node communication distance was 200 m. Sink node position was set according to actual situation. The network deployment results are shown in Figure 14. As shown in Figure 14, all nodes (a total of eight) were correctly deployed in the corresponding area, enabling full coverage monitoring. The distances between nodes and boundaries were greater than 2 m. There were no communications silos, and the minimum connectivity number of the network nodes was two, the maximum was six, the average network connectivity number was 4.25, and the maximum connectivity angle was almost 180°. The deployment fully meets the requirements of full coverage and robust connectivity of crop growth information monitoring.

### 6.3. WSN Deployment on Natural Farmland

**Figure 15:**
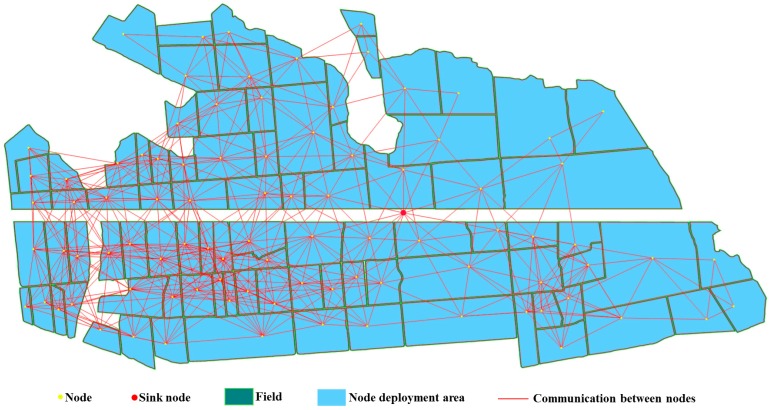
The WSN deployment on natural farmland located in Nanjing, Jiangsu Province, China. The communication distance is 200 m, and the connectivity is two.

The natural farmland selected is located in Nanjing (Jiangsu Province, China), with an area of 63 hectares and a total of 90 natural fields. All parameters of WSN deployment were the same as that of Section 6.2, except for the sink node location. The deployment results are shown in Figure 15. As shown in Figure 15, all nodes were deployed in the corresponding fields, and the distances to the boundaries were greater than 2 m, there were no communications silos, the minimum connectivity number of network nodes was two, the maximum was 20, and the average connectivity number of network nodes was 10.64. According to these analysis, the WSN deployed can full coverage monitor crop growth information with robust connectivity.

### 6.4. Performance of the Deployment Method

To effectively test the performance of the WSN deployment method of this paper, the object farmland should have many plots, so the farmland in Nanjing, shown in Section 6.3, was still selected to deploy WSN with different network connectivity, *k*, and different transmission distance of the node.

#### 6.4.1. The Deployment Performance for Different Value of *k*

**Figure 16:**
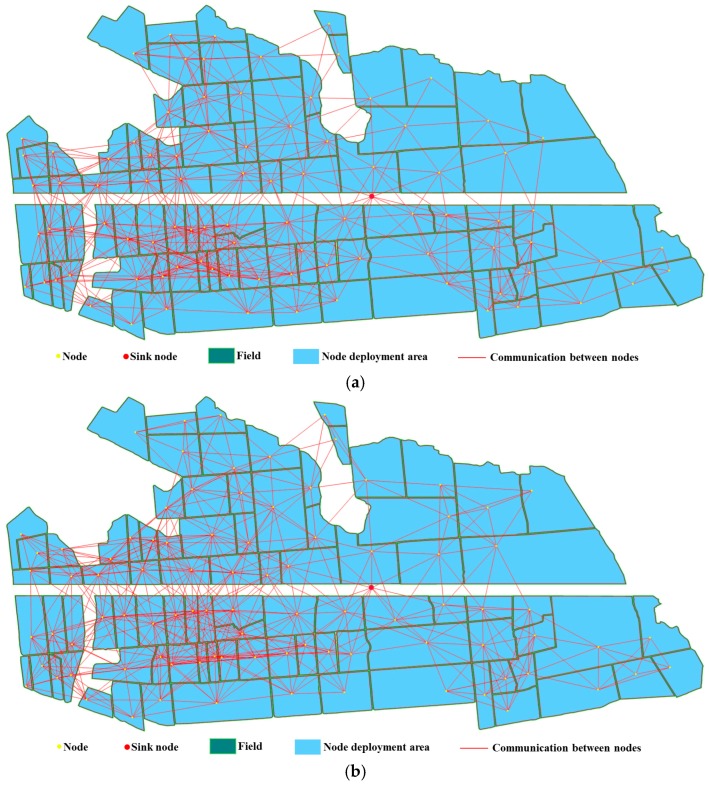
The WSN deployment on natural farmland located in Nanjing, Jiangsu Province, China. The communication distance is 200 m. (**a**) *k* = 3; (**b**) *k* = 4.

*k* was set to 2, 3, and 4, and the other parameters of deployment were the same as in Section 6.3. The deployment result when *k* was 2 is shown in Figure 15, and the results at 3 and 4 are shown in Figure 16. Figure 15 and Figure 16 show that all nodes are located in the corresponding plots, indicating that the networks provided full coverage.

**Figure 17:**
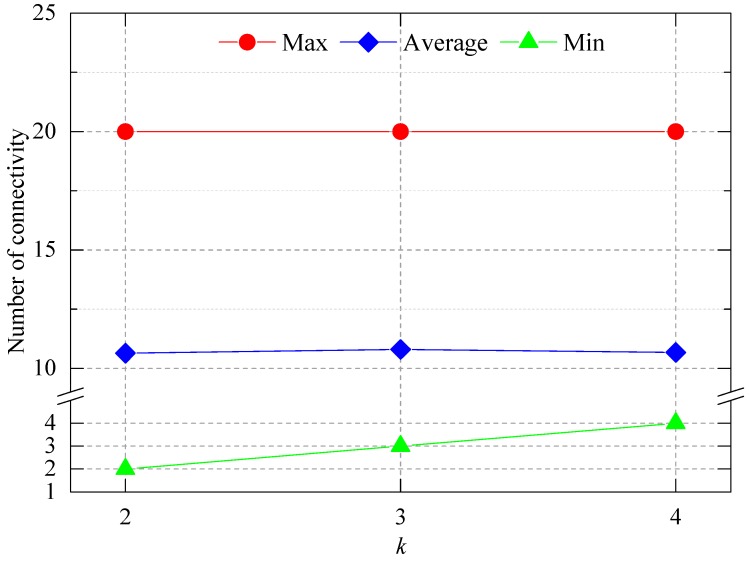
Network connectivity for different values of *k.*

The network connectivity situation was analyzed, including the maximum, minimum, and average connectivity when *k* is 2, 3, and 4. The data were the same values as shown in Figure 15 and Figure 16. The results are shown in Figure 17. As *k* increased, the maximum connectivity did not change, and the average connectivity changed only slightly. While, the minimum connectivity changes greatly and its value is exactly equal to *k*, which indicates that the deployed network satisfies the minimum connectivity requirement, and the network connection are robustness.

#### 6.4.2. Deployment Performance for Different Transmission Distances

The difficulty in *k*-connected WSN deployment has a strong relationship with the transmission distance of the node, the transmission distance of each node is here denoted by *d*. If *d* is large, a node could communicate with more other nodes, so it is easy to implement *k*-connected WSN. On the contrary, when *d* is very small, the *k*-connected WSN implementation becomes more difficult, because in this case, the nodes’ deployable area will be smaller to ensure the network is *k*-connected. Therefore, the connectivity of network nodes at different *d* can better test the performance of the deployment method.

**Figure 18:**
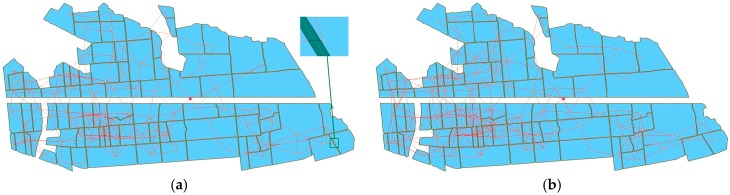

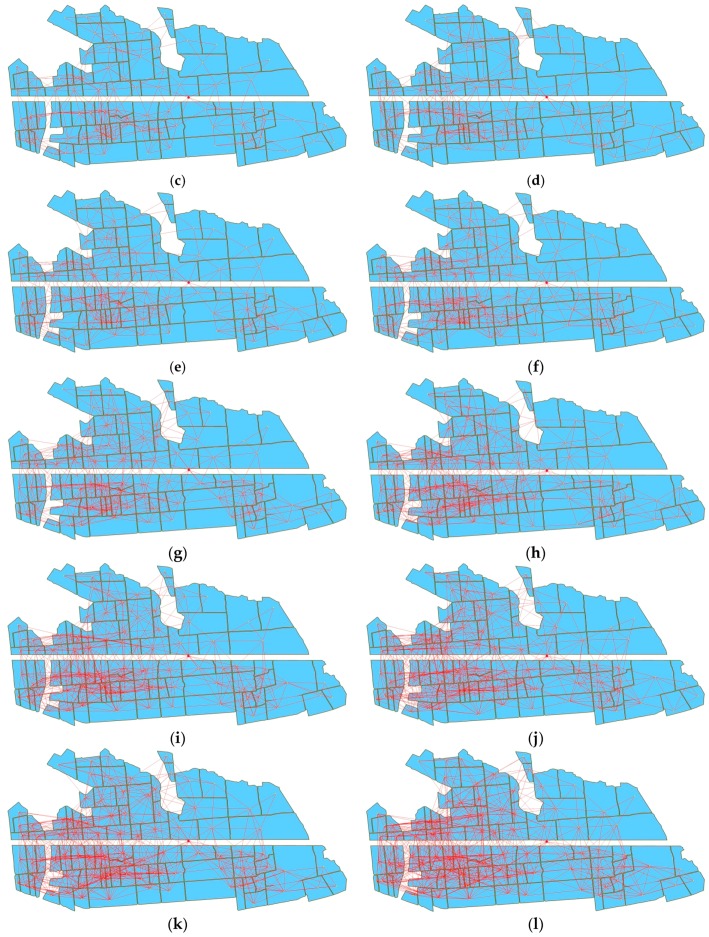
The WSNs deployed using method of this paper for different values of *d*. (**a**) *d* = 140 m; (**b**) *d* = 150 m; (**c**) *d* = 160 m; (**d**) *d* = 170 m; (**e**) *d* = 180 m; (**f**) *d* = 190 m; (**g**) *d* = 200 m; (**h**) *d* = 210 m; (**i**) *d* = 220 m; (**j**) *d* = 230 m; (**k**) *d* = 240 m; (**l**) *d* = 250 m.

**Figure 19:**
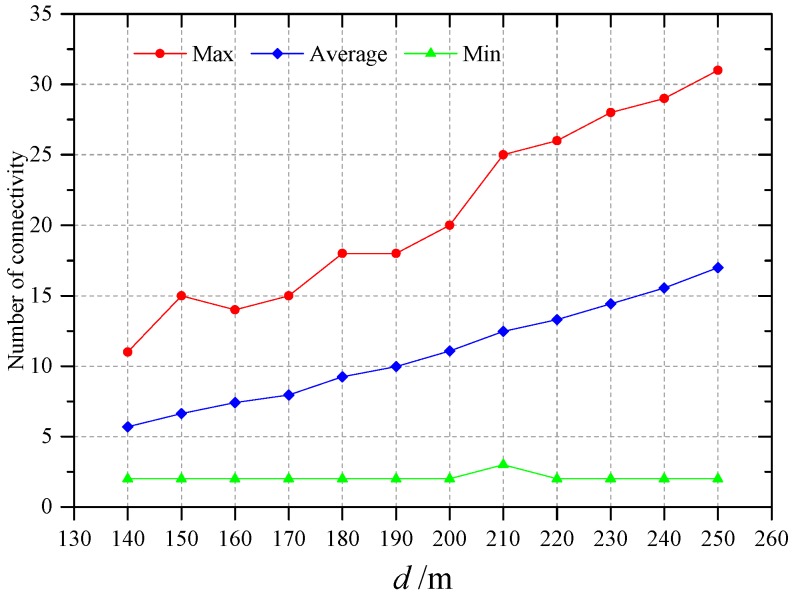
Network connectivity for different values of *d*.

The farmland located in Nanjing that is shown in Section 6.3 was still selected as the target area to deploy WSN. The *d* was set from 140 m to 250 m with interval of 10 m. In practical applications, the stability of network transmission can be guaranteed when *k* is 2. The reason is that, if the connectivity of each node is two and the probability of node failure is 1%, then the node information cannot be transferred to the sink node when the both neighbor nodes have failed. However, the probability of this happening is only one in 10,000. Therefore, the *k* was set to 2 in this section. The deployed WSNs by method of this paper are shown in Figure 18. The nodes of WSNs are all located in the corresponding plots, the connectivity of all WSNs are shown in Figure 19.

As shown in Figure 19, the maximum and average connectivity of nodes increase with the increase of *d* in a linear fashion, but the trend of maximum connectivity showing fluctuations, while the trend of average connectivity is very smooth. The minimum connectivity does not change with the change of *d*. It is always two, except when *d* is 210 m. Here, the reason why the minimum connectivity does not change with *d* should be related to the size and spatial position of farmland plots chosen for network deployment in this paper. When deployed on other farmland, the minimum connectivity may increase as *d* increases. Because the minimum connectivity of all networks deployed is greater than or equal to two, the networks deployed are all meet the *k*-connectivity requirement of WSN.

#### 6.4.3. Compared with Regular Grid Patterns Deployment Mothds

The number of nodes needed by deployment method of this paper was compared with those needed by three deployment methods that used regular patterns, namely, hexagons, squares, and triangle patterns. The number of nodes needed was calculated based on area per node (APN) and the farmland area. The maximal APNs of the three regular patterns were computed by using the method proposed by Bai et al. [81]:

(10) $$\gamma_{\max}^{Hex}=\frac{3}{4}\sqrt{3}{\left(\min\left\{R_{s},R_{c}\right\}\right)}^{2}$$

(11) $$\gamma_{\max}^{Squ}=2{\left(\min\left\{R_{s},\frac{\sqrt{2}}{2}R_{c}\right\}\right)}^{2}$$

(12) $$\gamma_{\max}^{Tri}=\frac{3}{2}\sqrt{3}{\left(\min\left\{R_{s},\frac{\sqrt{3}}{3}R_{c}\right\}\right)}^{2}$$

**Figure 20:**
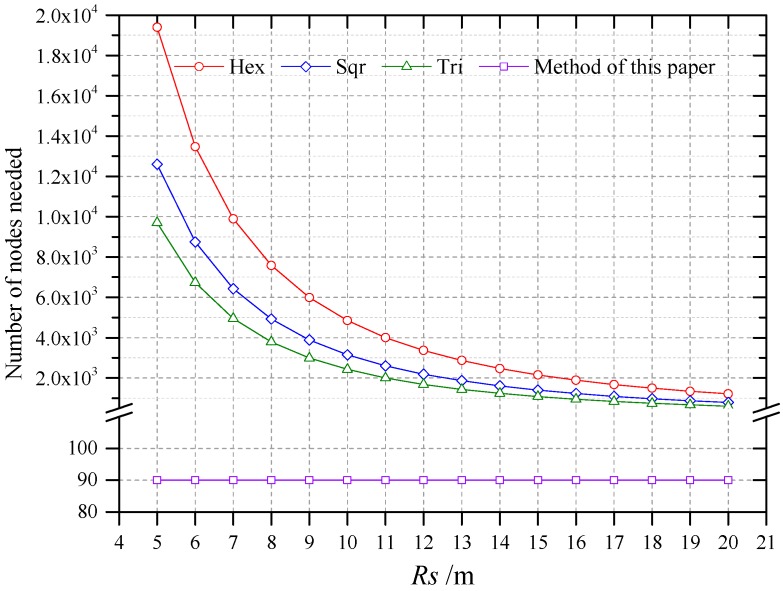
The number of nodes needed with different deployment methods.

$\gamma_{\max}^{Hex}$, $\gamma_{\max}^{Squ}$ and $\gamma_{\max}^{Tri}$ are the maximal APNs of regular hexagon, square and equilateral triangle, $R_{s}$ and $R_{c}$ are respectively the perception radius of node and the transmission distance of node. Here, $R_{c}$ was set to 200 m. $R_{s}$ was related of the sensors employed. The number of nodes needed of WSN deployed by method of this paper was equal to the number of plots. As shown in Figure 20, the number of nodes needed with different deployment methods. As illustrated in Figure 20, the numbers of nodes required by three deployment methods that use regular pattern are much more than that required by this paper’s method.

## 7. Conclusions

(1) Based on the characteristics of crop growth information monitoring, four criteria for WSN deployment were proposed. The method of WSN deployment with full coverage and *k*-connectivity for large-scale farmland was realized by using GA to meet the criteria. A large-scale WSN monitoring crop growth information was deployed in Rugao in Jiangsu Province, the transmission distance of node was 200 m. The study area was divided into sub-fields according to the spatial distribution of soil nutrients. Results showed that the network deployed using this method allowed for full-coverage monitoring of crop growth information, had no communications silos, and the minimum connectivity number of network nodes was two, the maximum was six, the average connectivity number of network nodes was 4.25, the network fully met the actual needs of agricultural production. A natural farmland with 63 ha, 90 plots, located in Nanjing in Jiangsu Province was selected to deploy WSN, the transmission distance of node was 200 m. The network deployed was full coverage and no communication silos. The minimum, maximum and average connectivity was 2, 20, and 10.46, respectively. The number of nodes needed was compared with those needed by three deployment methods that used regular patterns, namely, hexagons, squares, and triangle patterns. We found that these methods needed more nodes than the method described in this paper.
(2) A section of farmland of 63 ha was selected. It was divided into 90 plots as the WSN deployment area, located in Nanjing in Jiangsu Province. The connectivity of WSNs deployed by method of this paper was studied when the transmission distance was 200 m and the requirement network connectivity was 2, 3, and 4. The results showed that all WSNs were full coverage and no communication silos. In the case where the transmission distance is fixed, with the increase of requirement network connectivity, the maximum connectivity does not change, and the average connectivity changes only slightly. While, the minimum connectivity changes greatly and its value is equal to network connectivity required, indicate that the connectivity of WSNs deployed were robustness.
(3) A section of farmland of 63 ha, 90 plots was selected as the WSN deployment area, which located in Nanjing in Jiangsu Province. The connectivity of WSNs deployed by method of this paper was studied when the required network connectivity was two, and the transmission distance was from 140 m to 250 m at 10 m intervals. The results showed that, all WSNs deployed were full coverage and no communication silos. The minimum connectivity did not change with the change of transmission distance, the cause of the phenomenon may be related to the size and spatial position of farmland plots chosen for network deployment. When deploying on other farmland, the minimum connectivity may increase as transmission distance increases. The average connectivity increase linearly with the increase of transmission distance.

## Figures and Tables

**Table 1:** AHP scale.

Important Scale	Definition	Explanation
1	Equal importance	Two elements contribute equally
3	Moderate importance	One element is slightly favored over the other
5	Strong importance	One element is strongly favored over the other
7	Very strong importance	An element is very strongly favored over the other
9	Extreme importance	One element is extremely strongly favored over the other
2, 4, 6, 8	Between scales	
